# Safety net program participation and churning during the COVID-19 pandemic: a longitudinal analysis of low-income California families

**DOI:** 10.1016/j.ssmph.2026.101932

**Published:** 2026-05-08

**Authors:** Sydney Dougan, Kaitlyn E. Jackson, Rita Hamad, Wendi Gosliner, Kristina Dang, Elise Sheinberg, Lia C.H. Fernald

**Affiliations:** a Division of Community Health Sciences, School of Public Health, University of California Berkeley, Berkeley, CA, USA; b Department of Social & Behavioral Sciences, Harvard School of Public Health, Boston, MA, USA; c Nutrition Policy Institute, University of California, Division of Agriculture and Natural Resources, Oakland, CA, USA; d Leonard Davis School of Gerontology, University of Southern California, Los Angeles, CA, USA; e Department of Nutrition, Harvard School of Public Health, Boston, MA, USA

**Keywords:** Supplemental nutrition assistance program (SNAP), Special supplemental nutrition program for women, Infants, And children (WIC), Medicaid, Longitudinal analysis, Health equity, Social determinants of health

## Abstract

**Objective:**

Studies of participation in US safety net programs consistently find improvements in health equity, yet few have measured multi-program participation over time, or linked these trajectories to health during the COVID-19 pandemic. This study characterizes longitudinal participation phenotypes in SNAP, WIC, and Medicaid and examines their associations with mental and physical health.

**Methods:**

We used longitudinal data from a cohort of low-income families (n = 361, 2019–2023) and applied sequence analysis (Hamming distance) and cluster analysis to identify distinct trajectories of program use. We then characterized sociodemographic profiles across clusters and applied regression analyses to estimate associations between the safety net use phenotypes and self-reported health.

**Results:**

We identified six unique trajectories of participation. While program use increased initially (2019-2021), it was followed by declines and “churning” (cyclical loss/gain) in 2022-2023. The most prevalent clusters were “all programs” (25%) and “rapid churning” (22%). Consistent with the hypothesis that administrative burdens harm health, participants with stable, full participation (“all programs”) had consistently lower prevalence of adverse health outcomes compared to other clusters. Vulnerable groups (lower-income, racial/ethnic minorities) were disproportionately represented in high-churn clusters.

**Conclusion:**

Administrative volatility and “churning” were common, and results suggest that stability in coverage is a key driver of health equity. Policymakers should prioritize specific administrative safeguards, such as cross-program coordination, data sharing, and recruitment strategies to support vulnerable groups.

## Highlights

• We examined longitudinal trajectories in multi-program use from 2019 to 2023.
• We found evidence of safety net program churning, especially for SNAP.
• We found differences in demographic characteristics and health across safety net trajectories.
• We illuminate potential barriers to accessing multiple safety net programs for vulnerable groups.

## 1 Introduction

During the COVID-19 pandemic, US safety net programs underwent significant changes to counteract widespread economic upheaval in response to the crisis (Chiang et al., 2025). For example, the Supplemental Nutrition Assistance Program (SNAP), which provides monthly benefits to low-income households to purchase food, began providing more generous monetary benefits to low-income households for food (Jackson et al., 2024), and the Special Supplemental Nutrition Program for Women, Infants, and Children (WIC), a program for low-income pregnant women and families, removed in-person requirements and provided more funding for fruits and vegetables (Vasan et al., 2021). Medicaid, the US safety net health insurance program for people with low-income, waived recertification altogether (Schpero & Ndumele, 2022). Expanded SNAP and WIC benefits reduced anxiety and food insecurity (Bryant & Follett, 2022; Chaney et al., 2024; Jackson et al., 2024; Tsai, Anderson, et al., 2024), and the changes in Medicaid improved continuity of health coverage (Nelson et al., 2023). Additionally, between 2020 and 2023, SNAP enrollment increased from 39 to 42 million households (U.S. Department of Agriculture Food and Nutrition Service, 2025a), and Medicaid increased from 65 to 87 million (Desliver, 2025). Participation in WIC, which had been declining in previous years, remained stable at ∼6.2 million individuals (U.S. Department of Agriculture Food and Nutrition Service, 2025b).

Although program expansions increased costs to the federal government, there were longer term public cost savings (Berkowitz et al., 2017). In spite of these long-term efficiencies, the current U.S. federal government has prioritized immediate reductions in expenditures. Given the need to manage the national debt following the high cost of the October 2025 government shutdown, federal policy has aimed to offset this cost by restricting most benefits. These measures, often framed as necessary in the current deficit environment, utilize stricter administrative hurdles to generate short-term government cost savings.

As a consequence, gaps in program take-up (i.e., participation among eligible) remain a critical concern, particularly among more vulnerable groups (e.g., low-income people, racial/ethnic minorities) (U.S. Department of Agriculture Food and Nutrition Service, 2023; Jackson et al., 2025; Tsai, Yeb, et al., 2024). Since safety net programs are often administered by distinct government agencies, there are few analyses of longitudinal patterns in participation across multiple safety net programs. Even less is known about how patterns of multi-program participation varied among key subgroups or by health status, which is of particular importance given that helping individuals maintain access to programs they are eligible for improves their health and saves money for the federal government (Berkowitz et al., 2017).

To understand these dynamics, we draw upon the Administrative Burden Framework, which conceptualizes the costs that people face when interacting with the government. These burdens include learning costs (understanding eligibility), compliance costs (completing paperwork), and psychological costs (stress, stigma) (Herd & Moynihan, 2019). Prior to the pandemic, foundational research documented that high compliance costs drove churning, e.g., the cyclical loss and regain of benefits across SNAP and Medicaid, often resulting in coverage gaps that harmed health (Currie & Grogger, 2001; Mills et al., 2014; Sommers, 2016). In the context of the COVID-19 pandemic, there is a crucial question about how these burdens shifted, and how they relate to health outcomes. Answering these questions is critical for evaluating the resilience of the safety net.

We aimed to address these knowledge gaps and expand on the emerging literature related to cross-program participation within a longitudinal cohort of low-income California families, using sequence analysis to illuminate trajectories in multi-program use across five years (Brzinsky-Fay and Kohler, 2010; Raab & Struffolino, 2022). We also characterized sociodemographic profiles and self-reported health by program use trajectories. This study provides critical evidence to inform ongoing policymaking to improve cross-program coordination, data sharing, and recruitment strategies so that those facing economic disadvantages can continue to access programs to support health equity routinely as well as during future crises.

## 2 Methods

### 2.1 Study population

Data were drawn from the Accessing California Communities’ Experiences with Safety Net Supports (ACCESS) Study, a longitudinal cohort of individuals in California with at least one child under the age of eight. Recruitment involved a convenience sample of individuals accessing services from community-based organizations (e.g., local WIC offices). Baseline surveys were conducted August 2020-May 2021 (n = 497), and follow-up surveys were conducted January-June 2023 (n = 380, 76% retention). These included questions about sociodemographic characteristics, safety net program participation, health indicators, and other information related to the pandemic. We restricted the sample to individuals who participated in both waves of ACCESS and who had data on self-reported receipt of SNAP, WIC, and Medicaid from 2019 to 2023 (n = 361) (see sample flowchart, eFig. 1). Further details about the ACCESS Study are described in the Supplement.

Study protocols were approved by the California Committee for the Protection of Human Subjects and the Institutional Review Board of the University of California, Berkeley. Informed consent was provided by all respondents.

### 2.2 Demographic characteristics

Sociodemographic characteristics collected during both waves included respondents’ self-reported race/ethnicity, age, partnership status, primary language, education, number of children, annual household income (USD), and employment status. Only baseline responses for these variables were included in the analysis.

### 2.3 Safety net program participation

Participants were asked about program participation for SNAP, WIC, and Medicaid for any member of their personal household (i.e., respondent, spouse, or child [ren]), regardless of eligibility. It is possible that participants reported no participation in programs because they were ineligible. Other publications have reported take-up (i.e., participation among eligible) among ACCESS participants (Hamad et al., 2022; Jackson et al., 2025; Tsai, Yeb, et al., 2024). For the purpose of sequence analysis (described below), we measured participation. The present analysis is limited to SNAP, WIC, and Medicaid due to their generally widespread use and because other pandemic safety net programs had ended or expired (e.g., the 2021 Child Tax Credit expansion). At baseline, program participation was self-reported for years 2019 and 2020, and at follow-up, for years 2021, 2022, and 2023.

### 2.4 Health outcomes

Self-reported health in the past 12 months was collected at baseline and follow-up using a 5-item Likert scale (Idler & Benyamini, 1997), which was dichotomized (fair/poor versus good/very good/excellent). We used the validated 10-item Center for Epidemiologic Studies Depression Scale (CESD-10) to assess respondents’ depressive symptoms over the past week, creating a binary variable with a cut point of ≥ 10 (Andresen et al., 1994). Food insecurity was measured using the 6-item USDA adult food security scale (U.S. Department of Agriculture Economic Research Service, 2025), creating a binary variable indicating whether respondents had experienced any level of food insecurity (i.e., scores ≥ 1) within the past 30 days. At follow-up only, we assessed anxiety using the validated 2-item Generalized Anxiety Disorder scale (GAD-2), creating a binary variable using the score cut point of ≥ 3 (Kroenke et al., 2007).

### 2.5 Statistical analyses

We first tabulated baseline demographic characteristics and self-reported health for the overall sample, and calculated annual participation rates by program and year, regardless of eligibility or participation in other programs.

We used sequence analysis to characterize trajectories of program participation in SNAP, WIC, and Medicaid of participants across the study period (Brzinsky-Fay and Kohler, 2010; Chiang et al., 2025). Sequence analysis is able to capture program participation at each time point (year) in our study period, measuring how participation in each of our 8 states changed over time by quantifying transitions between each state (eTable 1), calculating the substitution costs, and quantifying the pairwise dissimilarities between sequences. For each year (2019 to 2023), participation was captured as one of eight possible states: [1] no programs, [2] SNAP only, [3] WIC only, [4] Medicaid only, [5] SNAP, WIC, [6] SNAP, Medicaid, [7] WIC, Medicaid, and [8] all programs. The “cost” to transform each trajectory into another was calculated using the Hamming Distance method, which emphasizes the timing of states and uses substitution costs between states, rather than insertion or deletion costs (insertions or deletion costs would insert/delete patterns from the sequence, thereby shifting the entire sequence) (Raab & Struffolino, 2022). Higher substitution costs are assigned for rare transitions, and lower substitution costs are assigned for frequent transitions. Costs ranged from a minimum value of 0 (i.e., no transition), to a maximum value of 2 (i.e., the value of an unobserved transition).

We conducted cluster analyses using the standard technique Partitioning Around Medoids (PAM), a process which selects representative sequences (“medoids”) and creates clusters by assigning similar sequences to these medoids using the pairwise dissimilarities (eTable 1) between sequences (Raab & Struffolino, 2022). To interpret identified clusters, we generated cluster quality indicators to determine which cluster solutions best fit the observed data (eTable 2). We assessed the number of clusters which produced maximum cluster quality metrics stabilized, but not yet “leveled out”. Upon this assessment, we supplemented the top cluster quality solutions (5-7 clusters) with expert judgment. Based on cluster size and interpretability, our main analysis evaluated the six-cluster solution (Raab & Struffolino, 2022).

To describe clusters, we tabulated baseline demographic characteristics and self-reported health at by cluster, then measured associations between cluster trajectories and health at follow-up, including both unadjusted and adjusted models. In these models, we included indicator variables for all five clusters. In adjusted models, inclusion of individual-level covariates enabled us to examine how health outcomes differ across clusters within levels of sociodemographic characteristics (i.e., to provide covariate-standardized contrasts) (see Supplement). We also used crude (unadjusted) models to descriptively contrast the clusters.

## 3 Results

Demographic characteristics and self-rated health are listed in Table 1, and annual participation rates for SNAP, WIC, and Medicaid are reported in eFig. 2.

**Table 1:** Demographic characteristics and self-reported health, overall and by cluster participation.

**Demographic characteristics**	Overall (N = 361)	Cluster 1. Rapid churning (N = 83)	Cluster 2. SNAP, Medicaid (N = 41)	Cluster 3. All program (N = 86)	Cluster 4. Lost WIC (N = 30)	Cluster 5. Medicaid, WIC (N = 70)	Cluster 6. SNAP churning (N = 51)
	N (%) or mean (SD)						
Female	344 (95%)	78 (94%)	39 (95%)	81 (94%)	28 (93%)	67 (96%)	51 (100%)
Race/ethnicity							
Hispanic	203 (56%)	41 (49%)	20 (49%)	48 (56%)	8 (27%)	54 (77%)	32 (63%)
Black	76 (21%)	24 (29%)	4 (10%)	22 (26%)	9 (30%)	7 (10%)	10 (20%)
White	42 (12%)	12 (14%)	12 (29%)	4 (5%)	10 (33%)	2 (3%)	2 (4%)
Other	40 (11%)	6 (7%)	5 (12%)	12 (14%)	3 (10%)	7 (10%)	7 (14%)
Spanish as primary language	156 (43%)	38 (46%)	11 (27%)	37 (43%)	6 (20%)	40 (57%)	24 (47%)
Partnered	141 (39%)	34 (41%)	6 (15%)	32 (37%)	9 (30%)	37 (53%)	23 (45%)
Age (years)	32 (6)	34 (7)	34 (6)	31 (6)	34 (5)	31 (7)	31 (7)
Number of children	2 (1)	2 (1)	2 (1)	3 (1)	3 (1)	2 (1)	3 (1)
Household income (USD), 2019 a	21011 (15199)	25420 (16375)	12667 (8175)	16050 (13022)	14408 (13082)	27679 (14885)	23641 (15805)
% federal poverty level a	81 (61)	101 (69)	51 (33)	61 (49)	56 (56)	104 (62)	88 (61)
Some college or more	183 (51%)	42 (51%)	26 (63%)	39 (45%)	16 (53%)	33 (47%)	27 (53%)
Work status, 2019							
Full-time	132 (37%)	33 (40%)	12 (29%)	25 (29%)	11 (38%)	34 (49%)	17 (33%)
Part-time	106 (29%)	25 (30%)	14 (34%)	26 (30%)	7 (24%)	20 (29%)	14 (27%)
Not working	122 (34%)	25 (30%)	15 (37%)	35 (41%)	11 (38%)	16 (23%)	20 (39%)
**Self-reported health at baseline**							
Fair/poor self-rated health b	66 (18%)	16 (20%)	9 (22%)	19 (22%)	8 (27%)	7 (10%)	7 (14%)
Depressive symptoms c	170 (47%)	40 (48%)	28 (68%)	42 (49%)	12 (40%)	26 (37%)	22 (43%)
Food insecure e	170 (48%)	35 (43%)	23 (57%)	35 (41%)	11 (37%)	39 (57%)	27 (53%)
**Self-reported health at follow-up**							
Fair/poor self-rated health b	153 (42%)	32 (39%)	23 (56%)	34 (40%)	15 (50%)	27 (39%)	22 (43%)
Depressive symptoms c	161 (45%)	36 (43%)	27 (66%)	35 (41%)	14 (47%)	27 (39%)	22 (43%)
Anxiety symptoms d	260 (72%)	58 (70%)	36 (88%)	59 (69%)	21 (70%)	51 (73%)	35 (69%)
Food insecure e	202 (56%)	47 (57%)	25 (61%)	44 (51%)	15 (50%)	40 (57%)	31 (61%)

Data were drawn from the Assessing California Communities' Experiences with Safety Net Supports Study (N = 361).

Abbreviations: SNAP = Supplemental Nutrition Assistance Program; WIC = The Special Supplemental Nutrition Program for Women, Infants, and Children.

a Annual adjusted gross income as confirmed on tax return paperwork, or self-reported annual income among non-filers or those who did not present tax return paperwork at time of interview. Percent of federal poverty level calculated based on 2019 federal poverty thresholds based on household size and income.

b Self-rated health were asked relative to the past 12-months. Fair/poor health coded as 1 and good/very good/excellent coded as 0.

c Depressive symptoms were assessed using the 10-item Center for Epidemiologic Studies Depression Scale, assessing respondents' depressive symptoms over the past week. The binary variable represents scores ≥ 10, indicative of high risk of clinical depression.

d Anxiety symptoms were assessed at follow up only using the validated 2-item Generalized Anxiety Disorder scale. The binary variable represents scores ≥ 3, indicative of high risk for generalized anxiety disorder.

e Food insecurity status was assessed for the last 30 days using the 6-item USDA adult food security scale. The binary variable represents scores >0, experiencing “any level food insecurity.”

**Fig. 1:**
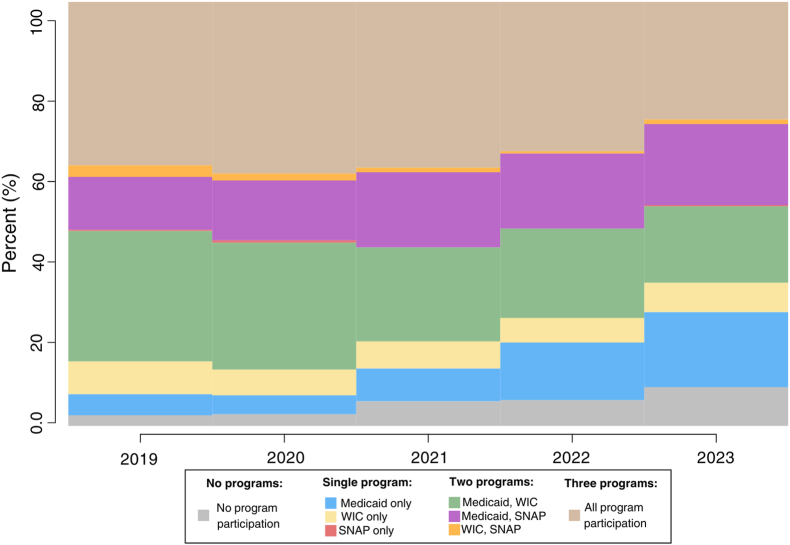
Proportion of participants in each safety net program participation “state” by each year. Data were drawn from ACCESS 2020-2023 (N = 361). Plot, generated from sequence analysis models, representing the proportion of participants in each participation category by year. Participation in single programs is indicated by primary colors (red, blue, and yellow), and participation in two programs is indicated by secondary colors, combining the primary program colors (orange, purple, green). Abbreviations: ACCESS=Assessing California Communities' Experiences with Safety Net Supports Study; SNAP = Supplemental Nutrition Assistance Program; WIC = The Special Supplemental Nutrition Program for Women, Infants, and Children.

“All program” participation described 39% of the sample in 2019, and decreased to 28% by 2023 (Fig. 1, Fig. 3). Few participants (2%) reported “no program” participation in 2019, which increased to 9% in 2023. The most common single program was “Medicaid only”, with 5% participation in 2019, which grew to 17% in 2023. “Medicaid, WIC” was the most common pair, with participation at 30% in 2019 and 18% in 2023; some participants transitioned to “all programs”, while others dropped one of the programs, or transitioned to another combination.

Cluster 1 (“rapid churning) represented 83 individuals and 22% of the sample. Cluster 2 (“SNAP, Medicaid”) represented 41 individuals and 11% of the sample. Cluster 3 (“all programs”) represented 86 individuals and 25% of the sample. Cluster 4 (“lost WIC”) represented 30 individuals and 8% of the sample. Cluster 5 (“Medicaid, WIC”) represented 70 individuals and 19% of the sample. Cluster 6 (“SNAP churning”) represented 51 individuals and 14% of the sample.

**Fig. 2:**
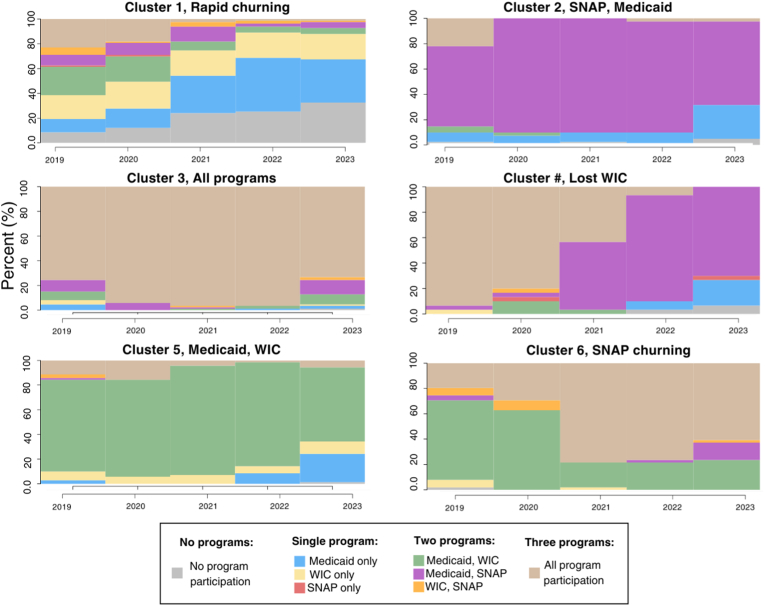
Proportion of participants in each safety net program participation “state” by each year, stratified by cluster. Data were drawn from ACCESS 2020-2023 (N = 361). Plots represent the proportion of participants in each participation category by year, stratified by cluster. Participation in single programs is indicated by primary colors (red, blue, and yellow), and participation in two programs is indicated by secondary colors, combining the primary program colors (orange, purple, green). Abbreviations: ACCESS=Assessing California Communities' Experiences with Safety Net Supports Study; SNAP = Supplemental Nutrition Assistance Program; WIC = The Special Supplemental Nutrition Program for Women, Infants, and Children.

Participants in Cluster 1 (“rapid churning”, 22%) experienced changes in program participation and lack of stability. Program participation peaked in 2019 and steadily decreased afterwards (Fig. 2, Fig. 4). Compared to other clusters, this cluster had a higher prevalence of Black participants (29%) and higher percentage of the Federal Poverty Level (FPL) (101%). Within this cluster across survey waves, the prevalence of fair/poor self-rated health was nearly double at follow-up (39%) compared to baseline (20%).

**Fig. 3:**
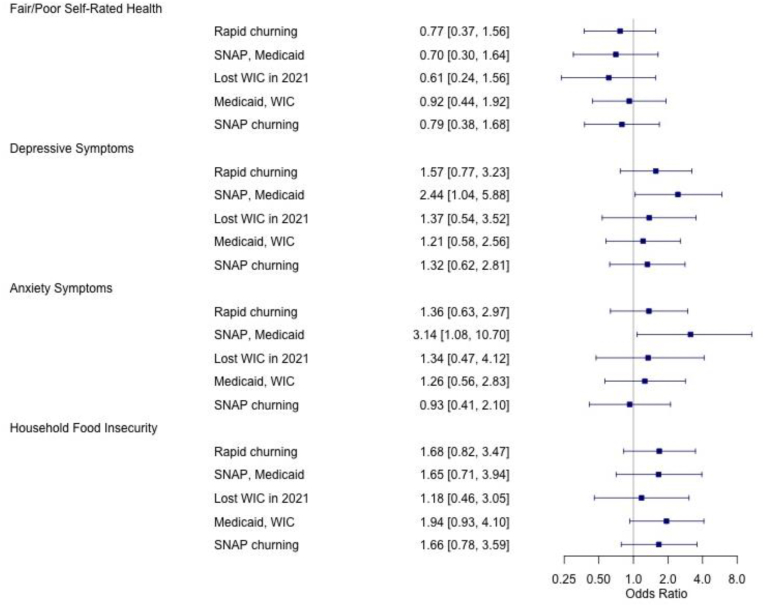
Associations between safety net trajectory clusters and health outcomes at follow up Data were drawn from ACCESS 2020-2023 (n = 361). Estimates represent odds ratios and 95% confidence internals from logistic regression models including indicator variables for each cluster, with the reference cluster being “all programs”, adjusted for race/ethnicity, age, partnered, 2021 income, Spanish as a primary language, and number of children in the household. Abbreviations: ACCESS=Assessing California Communities' Experiences with Safety Net Supports Study; SNAP = Supplemental Nutrition Assistance Program; WIC = The Special Supplemental Nutrition Program for Women, Infants, and Children.

**Fig. 4:**
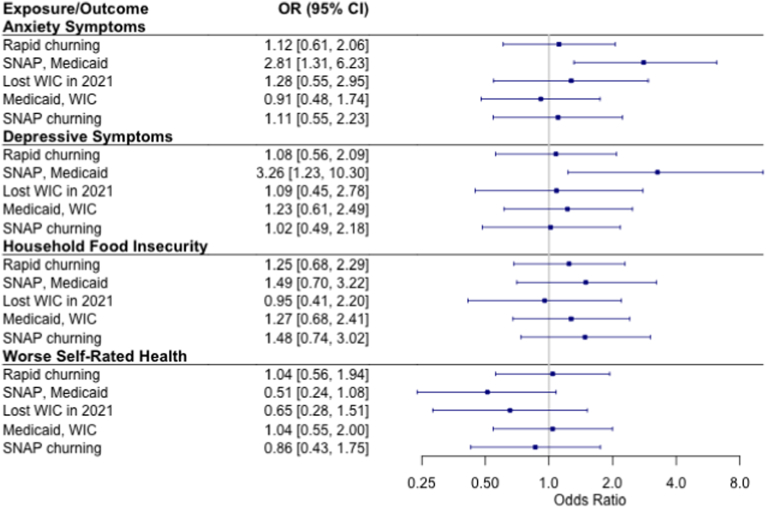
Associations between safety net trajectory clusters and health outcomes at follow up, unadjusted Data were drawn from ACCESS 2020-2023 (n = 361). Estimates represent odds ratios and 95% confidence internals from logistic regression models including indicator variables for each cluster, with the reference cluster being “all programs”, adjusted for race/ethnicity, age, partnered, 2021 income, Spanish as a primary language, and number of children in the household. Abbreviations: ACCESS=Assessing California Communities' Experiences with Safety Net Supports Study; SNAP = Supplemental Nutrition Assistance Program; WIC = The Special Supplemental Nutrition Program for Women, Infants, and Children.

Cluster 2 (“SNAP, Medicaid”, 11%) experienced generally stable participation in both programs across the period, although in 2023, a greater proportion of participants received Medicaid only. This cluster had the highest prevalence of White (29%) and the lowest prevalence of Black (10%) participants, and the lowest percentage of the FPL (51%) compared to other clusters. This cluster also had the highest prevalence of depressive symptoms and food insecurity at baseline (68% and 57%, respectively) and follow-up (66% and 61%, respectively), and the highest prevalence of anxiety symptoms at follow-up (88%) compared to other clusters. Within this cluster across survey waves, the prevalence of fair/poor self-rated health was more than double (22% versus 56%, respectively) at follow-up compared to baseline.

Cluster 3 (“all programs”, 25%) demonstrated more stable participation in all programs across the study period, especially from 2020 to 2022. Individuals were predominantly Hispanic (56%) and Black (26%) and the average % FPL was 61%, lower compared to some clusters. This cluster had lower prevalence of food insecurity at baseline (41%) and follow-up (51%), lower prevalence of fair/poor self-rated health (40%), and high depressive (41%) or anxiety (69%) symptoms at follow-up, compared to other clusters. Among this cluster across survey waves, the prevalence of fair/poor health doubled (22% versus 40% respectively), and food insecurity also increased (41% versus 51% respectively).

Cluster 4 (“lost WIC”, 8%) experienced generally stable participation in all programs up until 2021, after which participation in WIC declined, as well as participation in SNAP after 2022. “Lost WIC” was predominantly Black (30%) and White (33%) and also had generally lower percentage FPL (56%), compared to other clusters. This cluster had the lowest prevalence of food insecurity at baseline (37%) and follow-up (50%), compared to all other clusters. Among this cluster across survey waves, the prevalence of fair/poor health (27% versus 57%, respectively), depressive symptoms (40% versus 47%, respectively) and food insecurity (37% versus 50%, respectively) increased.

Cluster 5 (“Medicaid, WIC”, 19%) experienced generally stable Medicaid and WIC coverage across the study period. This cluster had the largest proportion of Hispanic (77%) and Spanish speaking (57%) participants, and higher percent FPL (104%), compared to other clusters. At baseline, “Medicaid, WIC” had a relatively lower prevalence of fair/poor self-rated health (10%) and depressive symptoms (37%), but the highest prevalence of food insecurity (57%), compared to other clusters. The prevalence of fair/poor self-rated health nearly quadrupled (10% versus 39%, respectively) and the prevalence of depressive symptoms slightly increased (37% versus 39%, respectively), across survey waves.

Cluster 6 (“SNAP churning”, 14%) experienced gaining and losing SNAP several times across the study period. “SNAP churning” was more Hispanic (63%) and had higher percent FPL (88%) compared to other clusters. This cluster had lower prevalence of fair/poor self-rated health (14%) compared to most other clusters. At follow-up the prevalence of other health outcomes was generally similar compared to other clusters. Among this cluster across survey waves, the prevalence of fair/poor self-rated health rose three-fold (14% versus 45%, respectively). Food insecurity also increased from 53% at baseline to 61% at follow-up.

We found slightly increased odds of depressive (odds ratio [OR]2.4, 95%CI:1.04,5.9) and anxiety symptoms (OR 3.1, 95%CI:1.08,10.7) associated with cluster 2 “SNAP, Medicaid” relative to those in “all programs” (Fig. 3). In unadjusted models, associations were similar in magnitude and direction to adjusted results (Fig. 4). We did not find evidence of statistically significant associations with health outcomes at follow-up for other clusters.

## 4 Discussion

This study is among the first to systematically describe individual-level, longitudinal participation in multiple safety net programs across the COVID-19 pandemic, overcoming substantial data limitations in linking administrative data for different US safety net programs which are administered by different government agencies. Key findings underscore a gradual increase in the use of multiple safety net programs between 2019 and 2021, followed by participation in only one or two programs for 2022 and 2023. Cluster and stratified analyses revealed associations between longitudinal trajectories in safety net program use and variables capturing socioeconomic and health status, with more marginalized groups (i.e., lower-income, Hispanic, Black) utilizing all programs, or Medicaid and WIC, but also experienced more safety net churning.

The decreased participation in programs from 2022 to 2023 is likely explained by expirations of pandemic-era safety net modifications, which varied by state (e.g. in California, SNAP expansions expired and Medicaid resumed eligibility redetermination in April 2023) (California Health Care Foundation, 2025). Changes in participation reported earlier in the pandemic may be due to changing eligibility of respondents such as a temporary increase in income or no longer being in the postpartum period for WIC, which we observed for the cluster “lost WIC”. Alternatively, respondents may still have been eligible but did not participate in programs for which they qualified; although most pandemic-era modifications were meant to expand coverage, ease application and/or eligibility requirements (e.g. reduce recertification requirements), and improve take-up, it is still possible that administrative barriers resulted in reduced participation.

Since self-reported participation in safety net programs can be unreliable due to recall bias (Celhay et al., 2021), it is also possible that participants erroneously reported program participation in earlier years. Given the rapidly changing policy landscape, and scattered communication by program, local/state, and federal government agencies during this period, it is plausible that respondents had trouble keeping track of what benefits they were receiving and when. It is also possible that program changes were not well recognized, understood, or utilized among our study sample of low-income families, further complicating participants' abilities to maintain (or keep track of) their coverage across the pandemic. A recent study found that many Americans thought they were uninsured throughout the pandemic even though they were eligible for Medicaid because they didn't realize they had Medicaid through the continuous coverage provision (Ding et al., 2024). These findings, coupled with the present analysis, suggest that while administrative burdens (e.g., program awareness) were reduced during the pandemic, they were not entirely eliminated.

Churning, repeated loss and gain of access to safety net programs, has been documented previously for Medicaid and SNAP (Mills et al., 2014; Nelson et al., 2023), and has been linked to high administrative compliance costs. Our findings suggest that churning was more common in SNAP and WIC and less common for Medicaid, likely due to the continuous coverage provision reducing compliance costs (Nelson et al., 2023). Viewed through the Administrative Burden Framework (Herd & Moynihan, 2019), this finding suggests that while policy changes reduced some barriers, the ‘learning costs' of navigating a shifting policy landscape and the remaining ‘compliance costs' of recertification continued to drive churn (Herd & Moynihan, 2019; Kenney et al., 2022), preventing millions of Americans from accessing critical benefits for which they were eligible. Not only is such churn damaging for households as they may temporarily lose access to needed benefits, but it is also costly for the government (Homonoff & Somerville, 2021). Our findings show that churning was consistently associated with poor health outcomes in our sample, as those in the cluster “SNAP churning” experienced the largest increase in the prevalence of fair/poor self-rated health from baseline to follow-up.

Participants in the clusters “rapid churning” and “SNAP churning” were more likely to be Hispanic, Black, or speak Spanish as their primary language compared to other clusters which experienced less churning. These findings are largely supported by a recent interrupted time series analysis of households with children in Massachusetts, which found that churning in SNAP in 2019 was most common among households with young children, and was disproportionately experienced by Black, Hispanic, and lower-income households (Kenney et al., 2022). We also found that a larger proportion of those participating in the “all programs” cluster and the “Medicaid, WIC” cluster were Black or Hispanic, which reinforces recent findings that WIC participation is generally higher among Hispanic and Black children (Guan et al., 2023). These findings are of particular importance given the worsening of preexisting health and economic disparities during the pandemic (Cai et al., 2021; Tai et al., 2020), and a growing body of research underscoring lower safety net program take-up among more vulnerable groups (Chiang et al., 2023; Gilbert et al., 2014; Hamad et al., 2022; Newman et al., 2011). One recent study found that only half of a sample of low-income caregivers of young children in California participated in multiple programs from 2019 to 2023, despite eligibility (Currie & Grogger, 2001). This same study also reported that program awareness, being lower income, younger, or not speaking English were factors associated with lower multi-program take-up (Currie & Grogger, 2001), factors identified in two other investigations regarding take-up of multiple US food and income assistance programs (Gilbert et al., 2014; Newman et al., 2011).

We found indications of worse self-reported health from baseline to follow-up, and those in clusters “all programs” and “Medicaid, WIC” had the best self-reported health, whereas “SNAP, Medicaid” had the worst self-reported health compared to other clusters. “SNAP churning” and “Medicaid, WIC” had the largest increase in fair or poor self-rated health from baseline to follow-up. We also found that cluster “SNAP, Medicaid” was associated with higher odds of depressive and anxiety symptoms compared to the “all programs” cluster. While statistical precision limited some comparisons given the small sample, the magnitude of the association for anxiety was substantial (OR 3.14). Moreover, a consistent pattern emerged across outcomes where the “all programs” trajectory was associated with the highest health vulnerability. Although we did not conduct a causal analysis, this finding could reflect individuals with worse mental health selecting into those programs, rather than the programs resulting in worsened mental health, which would be contrary to the prior literature (Jackson et al., 2024). This finding aligns with the theory that administrative burdens impose distinct psychological costs, including the stress of navigating benefit instability (“churning”) and the economic uncertainty it signals may directly exacerbate anxiety symptoms (Fernández-Viña et al., 2025; Gosliner et al., 2025). Notably, across all clusters, we found that the prevalence of anxiety symptoms at follow-up was higher compared to other health outcomes, consistent with prior work that depression and anxiety worsened during the pandemic nationwide (Cai et al., 2021). In the “SNAP, Medicaid” cluster, participants retained Medicaid but more appeared to lose SNAP as the study period progressed, so it is possible that this loss of SNAP was associated with poorer mental health trends in this group. Further research should employ quasi-experimental methods to better assess the causal direction for these findings.

A key limitation of our study is that ACCESS is a convenience sample, and we recruited participants from safety net program agencies. While this recruitment strategy ensures relevance, it biases the sample toward individuals who have already successfully navigated entry barriers. Thus, our findings are likely to represent a conservative estimate of the volatility faced by the broader less-connected, low-income population. Another limitation is that we were unable to restrict the sample to only those individuals eligible for every program (to capture take-up rather than participation) because this would have resulted in a prohibitively small sample. Consequently, our measure of non-participation is ambiguous because it captures both eligibility-driven reasons for leaving, such as income rising above thresholds, as well as procedural reasons for leaving, such as eligible households losing access due to administrative barriers or misinformation. Therefore, we cannot determine what proportion of the churning that we observe represents a failure of the safety net versus a positive economic transition.

Another limitation is that participants self-reported safety net participation and other characteristics, implying that our results may suffer from standard reporting biases; unfortunately, at this time it is logistically infeasible to link US administrative data with rich survey characteristics like those in the ACCESS Study. The risk of recall bias is heightened by the specific context of the pandemic with shifting eligibility rules, automatic renewals (e.g., for Medicaid), and the introduction of temporary benefits (e.g., P-EBT), which likely made it difficult for respondents to accurately distinguish between programs or recall the precise timing of their enrollment. However, methodological literature comparing survey data to administrative records generally indicates that respondents tend to underreport safety net participation due to stigma or memory errors (Meyer et al., 2015). Furthermore, while self-reports may not perfectly mirror administrative accounts of participation, they accurately reflect the lived experience of benefit instability, given that a participant's perception of coverage is a crucial component of administrative burden (Herd & Moynihan, 2019).

Finally, we acknowledge a risk of endogeneity in our regression models since our analyses prioritized descriptive characterization and were not intended to support causal inferences. Because the trajectory classification incorporates data from the entire study period including the time concurrent with follow-up health measurement, we cannot establish temporal precedence. For example, our dataset lacked information on disability status, which is relevant because disability is a driver of both continued program eligibility and lower self-rated health. For these reasons, associations between trajectories and health outcomes should be interpreted as characterizing the cumulative burden or safety net phenotype of individuals, rather than a strictly causal impact of the trajectory on health. Worse health in certain clusters may reflect selection into programs rather than program effects; future work could illuminate these relationships.

In spite of these limitations, this study has several strengths. We applied sequence and cluster analysis with longitudinal data, a novel approach in social science allowing for rich descriptions of trajectories of safety net participation for multiple programs over time. We collected data on multiple safety net programs and health outcomes, overcoming challenges in linking administrative data from multiple programs across distinct agencies.

Using sequence analysis, we examined patterns of safety net participation across multiple programs over time, reporting churning across multiple programs despite modifications intended to reduce administrative burdens. Program trajectories were associated with mental health, highlighting the need for future research to illuminate the associations between multiple safety net supports and health disparities. Our study adds to the existing literature by providing critical evidence to inform ongoing policymaking to prioritize continuous eligibility standards, automated data matching, and targeted recruitment strategies so that those facing economic disadvantages can access safety net support and achieve health equity.

Finally, although it is important to exercise caution with policy recommendations given that we are reporting observational research (Bann et al., 2025), our findings highlight the potential utility of specific administrative safeguards to address the observed disparities. First, the expansion of 12-month continuous eligibility, which is already standard in Medicaid for children and recently adopted for adults in several states, may offer a pathway to harmonize retention across SNAP and WIC to reduce the frequency of churning points. Second, efforts to maximize automated renewals using existing data sources could help mitigate the compliance costs that theoretically drive the instability identified in our analyses. Third, given the concentration of health risks within the “all programs” trajectory, agencies might consider data-sharing agreements where an individual presenting at a Medicaid office is automatically screened for WIC and SNAP support. Such mechanisms could help evolve the safety net from a system of separate silos into a proactive, integrated support structure.

## CRediT authorship contribution statement

**Sydney Dougan:** Writing – review & editing, Writing – original draft, Visualization, Methodology, Formal analysis. **Kaitlyn E. Jackson:** Writing – review & editing, Writing – original draft, Visualization, Project administration, Methodology, Investigation, Formal analysis, Data curation. **Rita Hamad:** Writing – review & editing, Writing – original draft, Supervision, Funding acquisition, Conceptualization. **Wendi Gosliner:** Writing – review & editing, Writing – original draft, Supervision, Funding acquisition, Conceptualization. **Kristina Dang:** Writing – review & editing, Visualization, Methodology, Formal analysis. **Elise Sheinberg:** Writing – review & editing, Visualization, Methodology, Formal analysis. **Lia C.H. Fernald:** Writing – review & editing, Writing – original draft, Supervision, Funding acquisition, Conceptualization.

## Ethics statement

Participants were interviewed with informed consent. Study procedures were approved by the California Committee of Human Subjects and the University of California, Berkeley Institutional Review board [CPHS 2019-10-12633; 2020-203-UC Berkeley]. The data presented in this study were collected and analyzed in accordance with the Declaration of Helsinki to ensure the protection and well-being of all study participants.

## Financial disclosure statement

The authors have no financial interests to disclose.

## Funding statement

This work was funded by the Robert Wood Johnson Foundation, the Tipping Point Foundation, the University of California Office of the President, and the Berkeley Population Center at the University of California Berkeley (National Institute for Child Health and Human Development of the National Institutes of Health, Award no. 5T32HD101364).

## Declaration of competing interest

The authors declare that they have no known competing financial interests or personal relationships that could have appeared to influence the work reported in this paper.

## Data Availability

The data that has been used is confidential.
