# Supplementary material for: Safety net program participation and churning during the COVID-19 pandemic: a longitudinal analysis of low-income California families
Source: SSM Popul Health. 2026 May 8;35:101932. doi: 10.1016/j.ssmph.2026.101932 (PMC13330658; doi:10.1016/j.ssmph.2026.101932)
Supplement: Multimedia component 1 [file mmc1.docx]

**Supplement**

**Study population**

Recruitment for the Assessing California Communities’ Experiences with Safety Net Supports (ACCESS) study involved a convenience sample of individuals accessing services from community-based organizations, such as safety net programs, social service agencies, or tax preparation services in California from August 2020 through May 2021. Participants were also asked to share study information with friends and family through a snowball sampling methodology. Digital recruitment materials included a link to an online eligibility screener to confirm EITC eligibility and provide consent and contact information before proceeding to the survey. The ACCESS study aimed to recruit an Earned Income Tax Credit-eligible sample at baseline, and therefore, inclusion criteria for ACCESS were: income within eligibility limits for the EITC based on marital status and number of dependents, and immigration status consistent with EITC eligibility rules.^1^ Prior work outlines recruitment methods utilized in ACCESS in greater detail.^2-6^

**Survey instrument**

Development of the survey instrument was informed by literature review, input from our Community Advisory Board, and our research team’s expertise in the field. Surveys were completed by trained members of the research team in both English and Spanish.

**Demographic characteristics**

Race/ethnicity category “other” included Asian, multi-race, and other race/ethnicity. These racial/ethnic categories were combined into one category for “other” due to small sample sizes. Respondents were asked to provide their tax return paperwork during their interview, and therefore, income is represented as adjusted gross income confirmed on tax return paperwork, or self-reported household annual income for non-filers or respondents who did not have their paperwork present.

**Safety net participation**

At baseline, participants who completed their survey from January-May 2021 were asked about safety net program participation “currently” (i.e., in 2021). Safety net program participation in 2021 was also collected at follow-up. For the purpose of the present analysis, we used 2021 participation from follow-up survey data in order to capture participation at any point through the entire year, whereas 2021 participation captured at baseline would have only included from January to the baseline interview date.

Annual participation rates for SNAP, WIC, and Medicaid, regardless of eligibility or participation in other programs, are reported in eFigure 2. Over 85% of respondents participated in Medicaid each year, whereas approximately half participated in SNAP, from 2019-2023. WIC participation fell from 80% in 2019 to 54% in 2023. Importantly, we report participation, rather than take-up (i.e., receipt among eligible), and therefore, observed declines in WIC may be due to respondents’ children aging out of the program over time.

**Health outcomes**

Respondents were asked to self-rate their health over the past year using a validated self-rated health item which has been consistently associated with markers of morbidity and mortality across diverse populations.^7^ Respondents were asked: “In the last 12 months, would you say your health in general has been excellent, very good, good, fair, or poor?”.^7^ We created a binary variable representing fair or poor health for the present analysis.

Respondents were also asked to complete the validated 10-item Center for Epidemiologic Studies Depression Scale (CESD-10) during their interviews to assess depressive symptoms over the past week.^8^ Scores could range from 0-30, with higher scores reflect increased depressive symptoms. We created a binary variable representing scores $\geq$10, indicative of significant depressive symptoms.^8^

Food insecurity status was measured using the 6-item USDA adult food security scale. Scores could range from 0-6, with higher scores representing lower food security (i.e., higher food insecurity).^9^ We created a binary variable classifying respondents with scores > 0, as “food insecure”, indicative of respondents who experienced any level of food insecurity within the past 30 days.

Lastly, at follow-up, we assessed anxiety using the validated 2-item Generalized Anxiety Disorder scale (GAD-2), measuring respondents’ anxiety symptoms over the previous two weeks.^10^ Scores could range from 0-6, with higher scores reflecting increased anxiety symptoms. Per standard protocol, we created a binary variable using the score cut point of $\geq$3, indicating of generalized anxiety disorder. This outcome was not collected at baseline.

**Sequence analysis**

Sequence analysis was conducted in R version 2023.06.1+524 using the *TraMineR* package.^11^

Using the eTable 1, the transition rate from “no participation” to “Medicaid” was 0.05, from “no participation” to “WIC” was 0.1; transitions from “no participation” to “WIC” occurred 2x the rate as “no participation” to “Medicaid”. More common transitions would correspond to a lower substitution cost, and conversely, rare transitions would correspond to a higher substitution cost. Thus, if a sequence was comprised of rare transitions, the total cost (summation of all the substitution costs) would be much greater (and more dissimilar) than a sequence comprised of the most frequent transitions.

**Cluster analysis**

We used the *WeightedCluster* package in R,^13^ to perform cluster analysis. To interpret identified clusters, we generated cluster quality indicators to determine which cluster solutions best fit the observed data (eTable 2). Generally, higher values of cluster quality metrics (with the exception of Hubert’s C) indicated that clusters showed reproducibility.^13^ We selected the top cluster quality solutions (5-7 clusters), selecting the 6-cluster solution following expert judgment based on cluster size and interpretability.^11-13^

**Secondary analyses**

We examined whether cluster assignment, as identified in cluster analysis, was associated with four health outcomes collected at follow up: self-rated health, depression symptoms, anxiety symptoms, and household food insecurity. We used logistic regression models to account for binary outcome variables. We carried out both adjusted and unadjusted models to descriptively contrast the clusters. Five binary variables indicating cluster assignment were included in regression models. The reference cluster was “all programs.”  Adjusted models also included individual-level covariates which may have confounded the relationship between cluster and health outcomes; race/ethnicity (reference non-Hispanic White), age $\geq$35, partnered, 2021 income $\geq$ $27,000 (i.e., mean income), Spanish as a primary language, and number of children in the household $\geq$3. Due to small sample size and issues surrounding multicollinearity, we were unable to include other covariates in regression models. The adjusted results are reported in Figure 3 and the unadjusted results are reported in Figure 4.

**Attrition analysis**

We conducted an attrition analysis examining baseline characteristics of the full study cohort who participated in the first wave of data collection (n=497) and those who participated in the second wave (n=380). We confirmed attrition was non-systematic. Results are reported in eTable 3.

**SUPPLEMENTAL TABLES**

**eTable 1.** Transition rate matrix representing substitution costs between safety net program trajectories

| **From Y-axis/To X-axis** | **No participation** | **Medicaid** | **WIC** | **Medicaid, WIC** | **SNAP** | **SNAP, Medicaid** | **WIC, SNAP** | **All programs** |
| --- | --- | --- | --- | --- | --- | --- | --- | --- |
| **No participation** | 0.8 | 0.05 | 0.1 | 0.02 | 0.0 | 0.08 | 0.0 | 0.0 |
| **Medicaid** | 0.09 | 0.7 | 0.01 | 0.05 | 0.0 | 0.1 | 0.0 | 0.04 |
| **WIC** | 0.1 | 0.0 | 0.6 | 0.1 | 0.01 | 0.03 | 0.02 | 0.06 |
| **Medicaid, WIC** | 0.0 | 0.08 | 0.04 | 0.6 | 0.0 | 0.02 | 0.01 | 0.2 |
| **SNAP** | 0.7 | 0.0 | 0.0 | 0.0 | 0.0 | 0.3 | 0.0 | 0.0 |
| **SNAP, Medicaid** | 0.04 | 0.2 | 0.0 | 0.0 | 0.0 | 0.7 | 0.0 | 0.08 |
| **WIC, SNAP** | 0.09 | 0.0 | 0.09 | 0.1 | 0.0 | 0.0 | 0.3 | 0.4 |
| **All programs** | 0.01 | 0.01 | 0.01 | 0.1 | 0.0 | 0.1 | 0.01 | 0.7 |
| Data were drawn from ACCESS 2020-2023 (N=361).  Abbreviations: ACCESS=Assessing California Communities’ Experiences with Safety Net Supports Study; SNAP = Supplemental Nutrition Assistance Program; WIC = The Special Supplemental Nutrition Program for Women, Infants, and Children. | | | | | | | | |

**eTable 2.** Cluster quality metrics from cluster analysis

| **Cluster number** | **Point Biserial Correlation** | **Hubert’s Gamma** | **Hubert’s Somers’ D** | **Average Silhouette Width** | **Weighted Average Silhouette Width** | **Calinski-Harabasz index** | **Pseudo R^2 a^** | **Pseudo F-statistic^b^** | **Pseudo R^2 c^** | **Hubert’s C** |
| --- | --- | --- | --- | --- | --- | --- | --- | --- | --- | --- |
|  |  |  |  |  |  |  |  |  |  |  |
| 1 | 0.4 | 0.5 | 0.5 | 0.3 | 0.3 | 61.2 | 0.2 | 98.2 | 0.2 | 0.2 |
| 2 | 0.6 | 0.7 | 0.7 | 0.3 | 0.3 | 72.8 | 0.3 | 132.5 | 0.4 | 0.2 |
| 3 | 0.7 | 0.7 | 0.7 | 0.4 | 0.4 | 64.1 | 0.4 | 121.5 | 0.5 | 0.1 |
| 4 | 0.7 | 0.9 | 0.9 | 0.4 | 0.4 | 67.7 | 0.4 | 148.9 | 0.6 | 0.1 |
| 5 | 0.7 | 0.9 | 0.9 | 0.4 | 0.4 | 62.6 | 0.5 | 146.8 | 0.7 | 0.1 |
| 6 | 0.7 | 0.9 | 0.9 | 0.4 | 0.4 | 59.7 | 0.5 | 143.4 | 0.7 | 0.1 |
| 7 | 0.7 | 0.9 | 0.9 | 0.4 | 0.4 | 57.4 | 0.5 | 139.1 | 0.7 | 0.1 |
| 8 | 0.7 | 0.9 | 0.9 | 0.4 | 0.4 | 53.9 | 0.6 | 133.1 | 0.8 | 0.1 |
| 9 | 0.7 | 0.9 | 0.9 | 0.4 | 0.4 | 51.6 | 0.6 | 127.7 | 0.8 | 0.1 |
| ^a^ Share of the discrepancy explained by clustering the solution  ^b^ Pseudo F-statistic computed from squared distances  c Share of the discrepancy explained by clustering solution, computed using squared distances | | | | | | | | | | |

**eTable 3.** Attrition analysis of baseline characteristics between first and second waves of data collection for ACCESS.

|  | **Full sample at first wave of data collection**  n= 497 | **Participants who remained in study for second wave**  n=380 |
| --- | --- | --- |
| Race/Ethnicity | | |
| Hispanic/Latinx | 287 (58%) | 209 (55%) |
| Non-Hispanic Black | 103 (21%) | 84 (22%) |
| White | 53 (11%) | 44 (12%) |
| Other | 54 (11%) | 43 (11%) |
| Marital Status | | |
| Not Partnered | 286 (58%) | 225 (59%) |
| Partnered | 211 (42%) | 155 (41%) |
| Age category | | |
| 18-34 | 328 (66%) | 240 (63%) |
| 35+ | 169 (34%) | 140 (37%) |
| Income category | | |
| $27,200 or less | 338 (68%) | 259 (68%) |
| More than $27,200 | 158 (32%) | 121 (32%) |
| Spanish primary language | 219 (44%) | 161 (42%) |
| Number of dependents | | |
| Two or less children | 311 (63%) | 241 (63%) |
| Three or more children | 186 (37%) | 139 (37%) |
| Data were drawn from ACCESS 2020-2023 (N=361).  Abbreviations: ACCESS=Assessing California Communities’ Experiences with Safety Net  Supports Study; SNAP = Supplemental Nutrition Assistance Program; WIC = The Special Supplemental Nutrition Program for Women, Infants, and Children. | | |

**SUPPLEMENTAL FIGURES**

**eFigure 1.** Sample flow chart


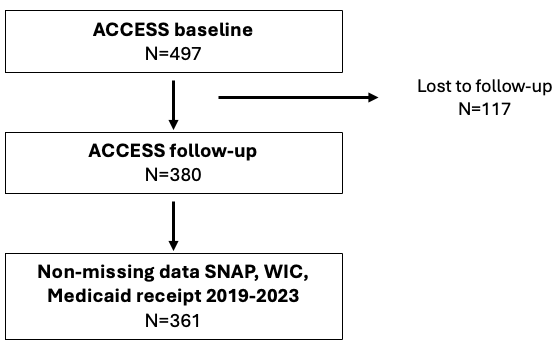


Abbreviations: ACCESS=Assessing California Communities’ Experiences with Safety Net Supports Study; SNAP = Supplemental Nutrition Assistance Program; WIC = The Special Supplemental Nutrition Program for Women, Infants, and Children.

**eFigure 2.** Participation in SNAP, WIC, Medicaid 2019-2023

Data were drawn from ACCESS 2020-2023 (N=361). Participation rates are reported regardless of eligibility status or concurrent participation in other programs.

Abbreviations: ACCESS=Assessing California Communities’ Experiences with Safety Net Supports Study; SNAP = Supplemental Nutrition Assistance Program; WIC = The Special Supplemental Nutrition Program for Women, Infants, and Children.

**eFigure 3.** Proportion of participants in each safety net program participation “state” by year.

*
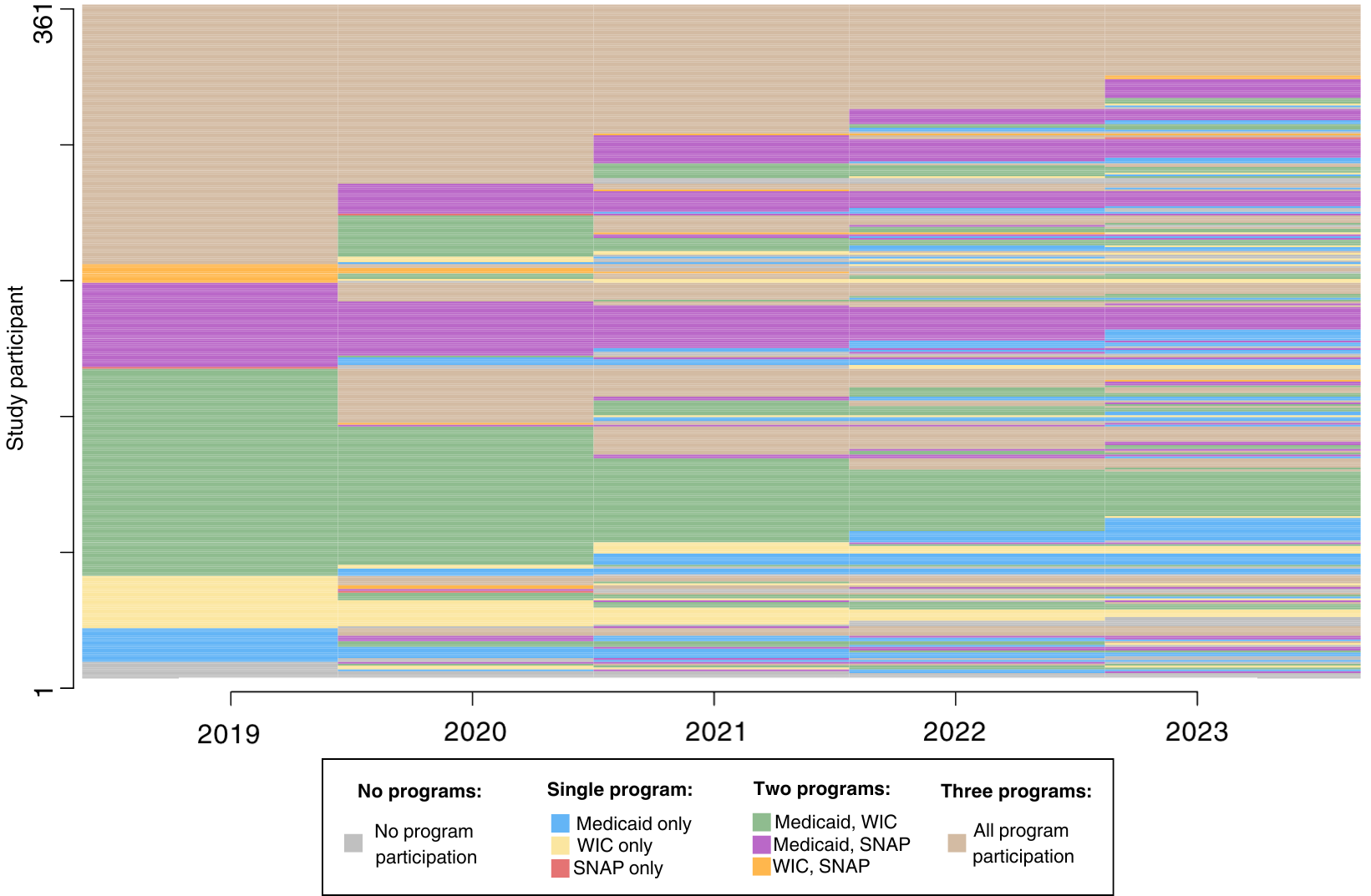
*

Data were drawn from ACCESS 2020-2023 (N=361). Index plot represents individual trajectories, with one row per individual. The 361 horizontal lines represent each individual ACCESS participants’ participation in program categories from 2019 to 2023. Participation in single programs is indicated by primary colors (red, blue, and yellow), and participation in two programs is indicated by secondary colors, combining the primary program colors (red and yellow to make orange, red and blue to make purple, blue and yellow to make green). Results were generated from sequence analysis models.

Abbreviations: ACCESS=Assessing California Communities’ Experiences with Safety Net Supports Study; SNAP = Supplemental Nutrition Assistance Program; WIC = The Special Supplemental Nutrition Program for Women, Infants, and Children

**eFigure 4.** Proportion of participants in each safety net program participation “state”, stratified by cluster


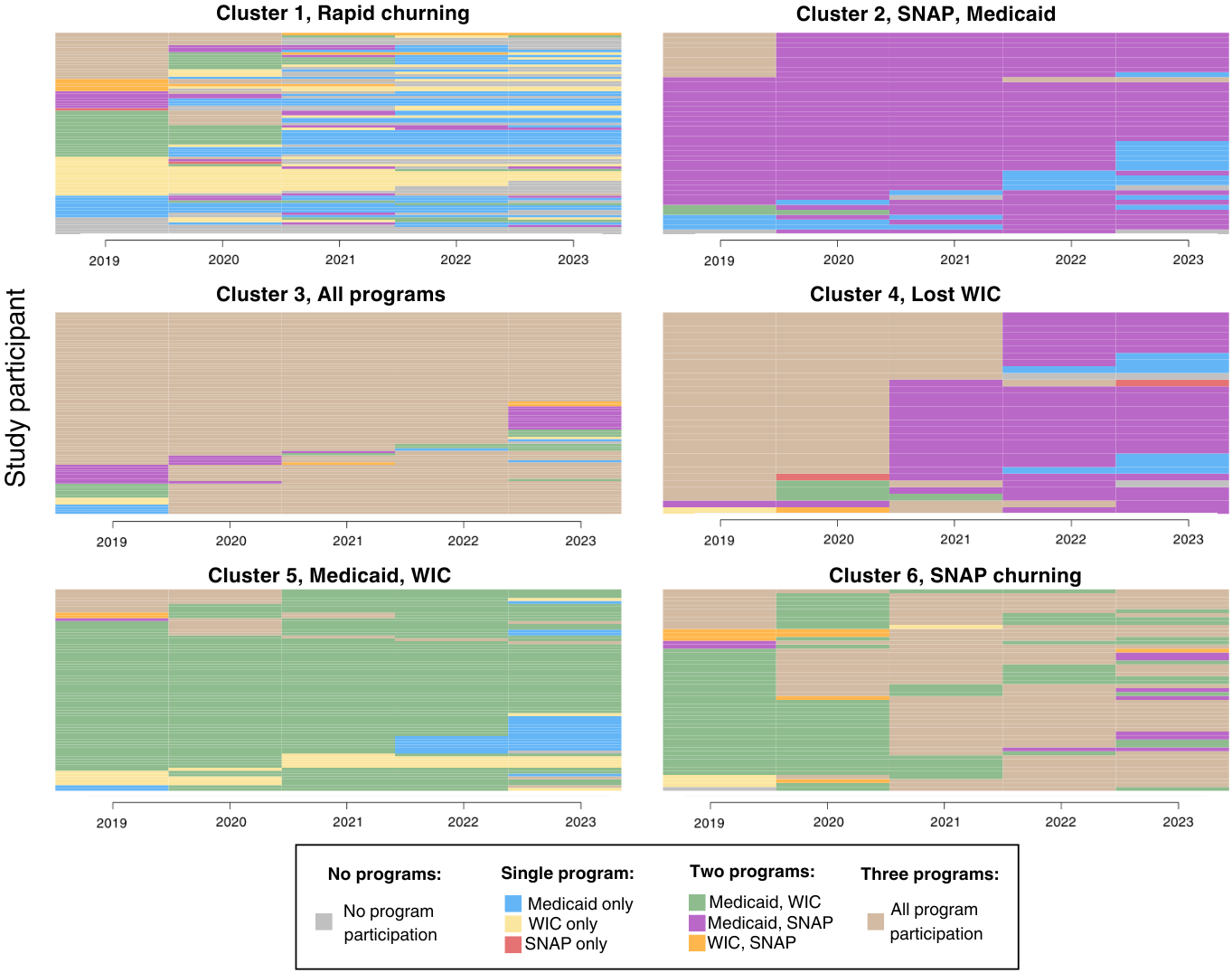


Data were drawn from ACCESS 2020-2023 (N=361). Each index plot represents individual trajectories, with one row per individual, stratified by cluster. Horizontal lines represent each individual participants’ participation trajectory in program categories from 2019 to 2023. Participation in single programs is indicated by primary colors (red, blue, and yellow), and participation in two programs is indicated by secondary colors, combining the primary program colors (red and yellow to make orange, red and blue to make purple, blue and yellow to make green)). Sample size differed by cluster. Cluster 1: N=83; Cluster 2: N=41; Cluster 3: N=86; Cluster 4: N=40; Cluster 5: N=70; Cluster 6: N=51.

Abbreviations: ACCESS=Assessing California Communities’ Experiences with Safety Net Supports Study; SNAP = Supplemental Nutrition Assistance Program; WIC = The Special Supplemental Nutrition Program for Women, Infants, and Children

**REFERENCES**

1. U.S. Internal Revenue Service. Who Qualifies for the Earned Income Tax Credit (EITC). 2023 7 February, 2024]; Available from: <https://www.irs.gov/credits-deductions/individuals/earned-income-tax-credit/who-qualifies-for-the-earned-income-tax-credit-eitc>

2. Brown EM, Fernald LCH, Hamad R, Hoskote M, Jackson KE, Gosliner W. Pandemic-related socioeconomic disruptions and adverse health outcomes: a cross-sectional study of female caregivers. BMC public health 2022;22(1):1893.10.1186/s12889-022-14287-2

3. Hoskote M, Hamad R, Gosliner W, Sokal-Gutierrez K, Dow W, Fernald LCH. Social and Economic Factors Related to Healthcare Delay Among Low-Income Families During COVID-19: Results from the ACCESS Observational Study. J Health Care Poor Underserved 2022;33(4):1965-1984.10.1353/hpu.2022.0148

4. Mooney AC, Jackson KE, Hamad R, Fernald LC, Hoskote M, Gosliner W. Experiences of distress and gaps in government safety net supports among parents of young children during the COVID-19 pandemic: a qualitative study. BMC public health 2023;23(1):1099

5. Pulvera R, Jackson K, Gosliner W, Hamad R, Fernald LC. The association of safety-net program participation with government perceptions, welfare stigma, and discrimination. Health Affairs Scholar 2024;2(1):qxad084

6. Tsai MM, Yeb J, Jackson K, Gosliner W, Fernald LCH, Hamad R. Understanding multi-program take-up of safety net programs among California families American Journal of Preventive Medicine Focus 2024;in press

7. Idler EL, Benyamini Y. Self-rated health and mortality: a review of twenty-seven community studies. J Health Soc Behav 1997;38(1):21-37

8. Andresen EM, Malmgren JA, Carter WB, Patrick DL. Screening for depression in well older adults: evaluation of a short form of the CES-D (Center for Epidemiologic Studies Depression Scale). Am J Prev Med 1994;10(2):77-84

9. U.S. Department of Agriculture Economic Research Service. Food Security in the U.S. - Survey Tools; 2025.

10. Kroenke K, Spitzer RL, Williams JB, Monahan PO, Löwe B. Anxiety disorders in primary care: prevalence, impairment, comorbidity, and detection. Ann Intern Med 2007;146(5):317-25.10.7326/0003-4819-146-5-200703060-00004

11. Gabadinho A, Ritschard G, Müller NS, Studer M. Analyzing and Visualizing State Sequences in R with TraMineR. Journal of Statistical Software 2011;40(4):1 - 37.10.18637/jss.v040.i04

12. Raab M, Struffolino E. Sequence analysis. Sage Publications; 2022.

13. Studer M. WeightedCluster library manual. A practical guide to creating typologies of trajectories in the social sciences with 2013;2013(24):33
